# Targeted drug delivery strategy: a bridge to the therapy of diabetic kidney disease

**DOI:** 10.1080/10717544.2022.2160518

**Published:** 2022-12-28

**Authors:** Xian Chen, Wenni Dai, Hao Li, Zhe Yan, Zhiwen Liu, Liyu He

**Affiliations:** a Department of Nephrology, The Second Hospital of Hebei Medical University, Shijiazhuang, Hebei, People’s Republic of China; b Department of Nephrology, Hunan Key Lab of Kidney Disease and Blood Purification, The Second Xiangya Hospital, Central South University, Changsha, Hunan, People’s Republic of China

**Keywords:** Diabetic kidney disease, diabetes mellitus, targeted drug delivery, nanoparticle, liposome

## Abstract

Diabetic kidney disease (DKD) is the main complication in diabetes mellitus (DM) and the main cause of end-stage kidney disease worldwide. However, sodium glucose cotransporter 2 (SGLT2) inhibition, glucagon-like peptide-1 (GLP-1) receptor agonist, mineralocorticoid receptor antagonists and endothelin receptor A inhibition have yielded promising effects in DKD, a great part of patients inevitably continue to progress to uremia. Newly effective therapeutic options are urgently needed to postpone DKD progression. Recently, accumulating evidence suggests that targeted drug delivery strategies, such as macromolecular carriers, nanoparticles, liposomes and so on, can enhance the drug efficacy and reduce the undesired side effects, which will be a milestone treatment in the management of DKD. The aim of this article is to summarize the current knowledge of targeted drug delivery strategies and select the optimal renal targeting strategy to provide new therapies for DKD.

## Introduction

Diabetic kidney disease (DKD) is the predominant complication of diabetes mellitus (DM). Approximately 21.3% of the DM patients accompany with chronic kidney disease (CKD) in China, and the incidence is increasing year by year (Zhang et al., 2016). DKD is the most common cause of renal failure all over the world, and about 50% of patients progressed to dialysis or transplant stage in the USA, which significantly elevates the risk for mortality (DeFronzo et al., 2021). Effective treatment options are in urgent need to postpone the disease progression. Except for the traditional agents, such as angiotensin-converting enzyme inhibitors (ACEI) and angiotensin II receptor blockers (ARB), sodium glucose cotransporter 2 (SGLT2) inhibition, glucagon-like peptide-1 (GLP-1) receptor agonist, mineralocorticoid receptor antagonists (MRAs) and endothelin receptor A inhibition have a beneficial impact on the control of the pathophysiological abnormalities (Michos and Tuttle, 2021; Zhou et al., 2021; Barrera-Chimal et al., 2022; Ravindran and Munusamy, 2022). In spite of this, the adverse reaction is unavoidable and the incidence of diabetic nephropathy has not declined, with the result that many patients still inevitably develop uremia. In this condition, novel treatment options with improved targetability, bioavailability, efficacy and safety are urgently needed.

Targeted drug delivery strategy is a rapidly developed method in which the specific bio-actives are transported with carrier system to the predetermined organ or cell. It enables the therapeutic agents specifically transfer and accumulate at the diseased sites and increases the local concentration of drugs and minimizes the side effects (Liang et al., 2021). In this process, three important components are included as follows: first, the specific drug moiety; second, the drug delivery carrier; third, the targeted cells, tissues or organs to be treated. There are two types of mechanisms for carrier-targeted drug delivery systems: slowing down the rate of drug release, or releasing in large quantities at the target site by adjusting for changes in pH, heat, or light (Ashique et al., 2021). The carrier, with the properties of nontoxicity, biodegradability, diminished immunogenicity and easy detection, is used to encapsulate the drug molecules for delivery to the target organs, while enhancing the solubility, permeability and bioavailability of the drug molecules (Su et al., 2015).

The kidney-targeted drug delivery systems were comprehensively raised by Haas in 2002 (Haas et al., 2002). In the following two decades, the related researches about targeted therapies for different kinds of kidney diseases and renal ultrastructure were published successively. Targeted drug delivery for DKD is one of the current research hotspots and may be a promising solution for the prevention and treatment of DKD. The purpose of this review is to comprehensively summarize the current knowledge of targeted drug delivery strategies for DKD from the perspective of the pathogenesis and the renal ultrastructure, in order to select the optimal targeting principles and potential drug carriers to provide new therapies for DKD.

## Diabetic kidney disease

DKD is one of the most severe microvascular complications of DM, which is often associated with cardiovascular disease and increased the risk of mortality in diabetic patients (Samsu, 2021; Fox et al., 2012). The estimated number of global DM patients has reached 463 million and the prevalence of DKD has obviously increased in the recent two decades (Saeedi et al., 2019). About 35 to 40% of type 1 or 2 diabetes patients will eventually develop DKD, which has a profound implication for global public health and socioeconomics (Yang et al., 2018).

### Pathogenesis of DKD

**Figure 1.**
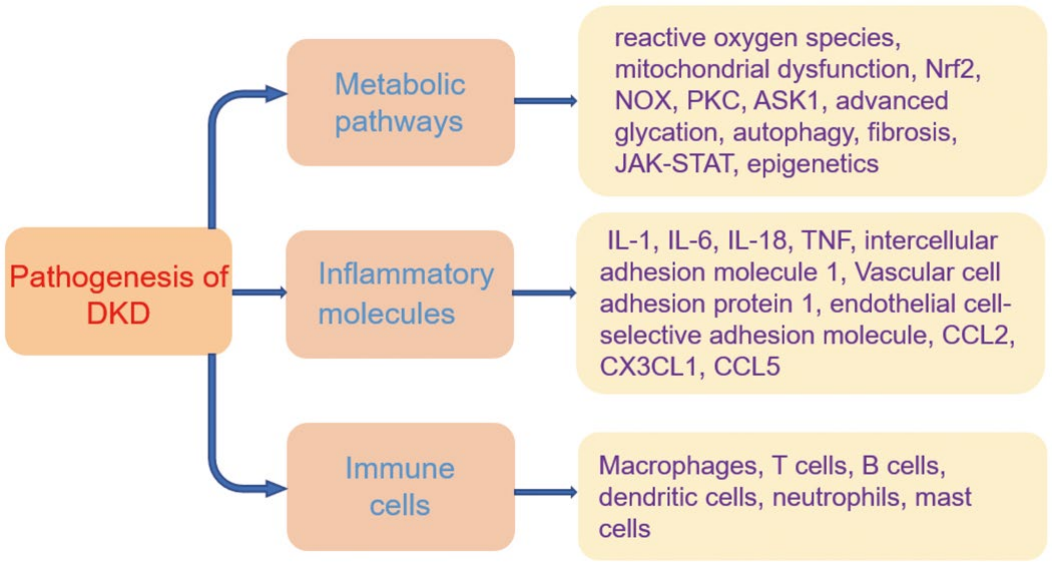
The potential pathogenesis of diabetic kidney disease (DKD). Nrf2, transcription factors; NOX, NADPH oxidase; PKC, protein kinase C; ASK1, apoptosis-signaling kinase 1; JAK-STAT, Janus kinase-signal transducer and activator of transcription; TNF, tumor necrosis factor.

The pathogenesis of DKD is complicated and involves plenty of different pathways (Figure 1). It is always regarded that DKD is a non-inflammatory disease caused by metabolism, but with the deepening of research, increasing evidence suggests that inflammation also participates in its pathogenesis. The metabolic pathways in DKD mainly include reactive oxygen species (Han et al., 2018), mitochondrial dysfunction (W Dai et al., 2021), transcription factors (Nrf2) (S Li et al., 2021), NADPH oxidase (NOX) (You et al., 2016), protein kinase C (PKC) (Yin et al., 2019), apoptosis-signaling kinase 1 (ASK1) (Tesch et al., 2015), advanced glycation (Sanajou et al., 2018), autophagy (Yang et al., 2021), fibrosis (Jha et al., 2022), Janus kinase-signal transducer and activator of transcription (JAK-STAT) (Brosius et al., 2016) and epigenetics (Kato and Natarajan, 2019). Increased expression of inflammatory cytokines, adhesion molecules, growth factors and chemokines, including IL-1, IL-6, IL-18, tumor necrosis factor, intercellular adhesion molecule 1, Vascular cell adhesion protein 1, endothelial cell-selective adhesion molecule, CCL2, CX3CL1, CCL5 and so on, are reported to be involved in the pathogenesis of DKD (Navarro-González et al., 2011). Immune cells, such as macrophages, T cells, B cells, dendritic cells, neutrophils and mast cells, are commonly observed in DKD patients (Moon et al., 2012; Klessens et al., 2017; Yang and Mou, 2017). These pathways are potential candidates for the DKD therapy. The deeper the research mechanism, the more precise the targeted therapy of diabetic nephropathy.

### Pathological changes of DKD

DKD was traditionally diagnosed by the development of persistent albuminuria and the followed progressive GFR decline, which was defined as the typical phenotype of DKD (National Kidney Foundation, 2012). However, several studies have reported that some DM patients without albuminuria progress to renal insufficiency, described as normoalbuminuric diabetic kidney disease (NADKD) (Q Dai et al., 2021; Jia et al., 2022). In a retrospective study, 32 out of 36 deceased diabetic kidney donors, presenting with non-albuminuria and an estimated GFR > 60 ml/min/1.73m^2^, showed histopathological lesions consistent with DKD, which indicated a high proportion of undiagnosed DKD (Comai et al., 2019). Therefore, the renal biopsy is of great importance in the diagnosis of DKD.

Glomerular basement membrane (GBM) thickening is considered as the earliest observed pathological feature in patients with DKD, which is appeared within 1–2 years after the onset of DM (Tervaert et al., 2010; Ponchiardi et al., 2013). Endothelial cells play an important role in the progression of DKD. With the development of DKD, the fenestrated ECs are decreased in diabetic patients, which correlates with albuminuria and the loss of GFR (Dou and Jourde-Chiche, 2019). Mesangial expansion, caused by Mesangial cells (MCs) enlargement and accumulation of glomerular matrix protein, is the most common renal pathological change in DKD (Reidy et al., 2014; Zhang et al., 2019). On the glomerular capillary side of MCs, without the surrounding of GBM and podocytes, drugs can be delivered to MCs for treating kidney diseases (Scindia et al., 2008). Podocytes are glomerular epithelial cells which contain 3 separate elements: cell body, extending processes and foot processes (Garg, 2018). Podocytes injury in DKD is induced by many compound factors, such as inflammatory reaction, mechanical stress, oxidative stress, renin angiotensin aldosterone system activation, TGF-β1 induction, and AGEs accumulation, and any part of the pathway is expected to be the target of DKD therapy (Kawanami et al., 2016). The renal tubules consist of the proximal tubules, collecting tubules and distal tubules. The morphological and functional changes of the renal tubules are involved in the pathogenesis and progression of DKD (Duan et al., 2021). Most renal tubular targeted systems are directed at the proximal tubules (Christensen et al., 2012).

### Management of DKD

The management of DKD mainly contains modification of lifestyle (smoking cessation, proper exercise, low-salt and low-protein diet and weight reduction), glucose control, blood pressure control and lipid profiles control. Some novel glucose-lowering agents have both glucose control and renal protective effects. SGLT2 inhibitor can inhibit the glucose reabsorption in proximal tubular and promote urinary glucose excretion. Empagliflozin, an SGLT2 inhibitor, can significantly reduce the cardiovascular events in type2 DM and delay the progression of kidney disease (Zinman et al., 2015; Wanner et al., 2016). Canagliflozin and dapagliflozin demonstrated the similar renal protection (Neal et al., 2017; Wiviott et al., 2019). GLP-1 receptor agonist and dipeptidyl peptidase-4 (DPP4) inhibitor are incretin hormone, released from the gastrointestinal tract, which can stimulate insulin secretion and keep glucose in a stable level. Recent trials have further illustrated the kidney benefits of GLP1 receptor agonist in DKD (Gerstein et al., 2021). DPP4 inhibitor can reduce the albuminuria level, but the renoprotective effect needs further research (Mosenzon et al., 2017). Mineralocorticoid receptor antagonists (MRAs) are a prospective therapeutic option in DKD because of the activation of mineralocorticoid receptor in podocytes, mesangial cells, inflammatory cells, fibroblasts, and vascular cells (Barrera-Chimal et al., 2022). Spironolactone and eplerenone, kinds of MRAs, can decrease albuminuria and ameliorate renal injury in DKD (Makhlough et al., 2014; El Mokadem et al., 2020). Although these new hypoglycemic drugs have satisfying effects in the treatment of DKD, some side effects, just as the urinary tract infection of SGLT2 inhibitor, the gastrointestinal reaction of incretin hormone, the hyperkalemia of MRAs, limit their application in some patients. As the impaired glomerular filtration and tubular damage in DKD, the conventional dosage of drug does not ensure the required amount of it get into the targeted cell. Kidney targeted drug delivery strategy may help to solve the above problems.

## Targeted drug delivery strategy

The development of new drugs usually fails in the final stage due to safety and efficacy, and the root causes are the high enrichment of off-target organs and the low enrichment of target organs, which lead to the dose-related toxicities and dose-limited efficacies. Targeted drug delivery strategy is designed to enhance the drug’s therapeutic efficacy through targeted distribution and to decrease the undesired toxicities and reduce the off-target effects. The three important parts in this process are drugs, delivery systems and the targeted organs or cells. The drug delivery system contains five different types: passive drug targeting, active drug targeting, cancer cell targeting, endothelial cell targeting and triggered drug release (Ozturk-Atar et al., 2018). Passive drug targeting systems rely on pharmacological or physicochemical factors to work and they do not have a specific targeting ligand to guide drugs or carriers to their target, so they need a prolonged circulation time in the blood in order to have enough drug amounts in target site (Hirsjarvi et al., 2011). Active drug targeting included both direct and indirect ways. The direct active targeting refers that the moieties on the drug or the drug carrier directly conjugate to the target cells or organs through a specific structure (Byrne et al., 2008). The indirect ways need a two-step strategy, just as the antibody-directed enzyme prodrug therapy or gene-directed enzyme prodrug therapy (Schellmann et al., 2010). Compared to the passive drug targeting, the active targeting can significantly increase the number of drugs that are delivered to the target cell (Ashique et al., 2021).

## Targeted drug delivery strategy in DKD

**Figure 2.**
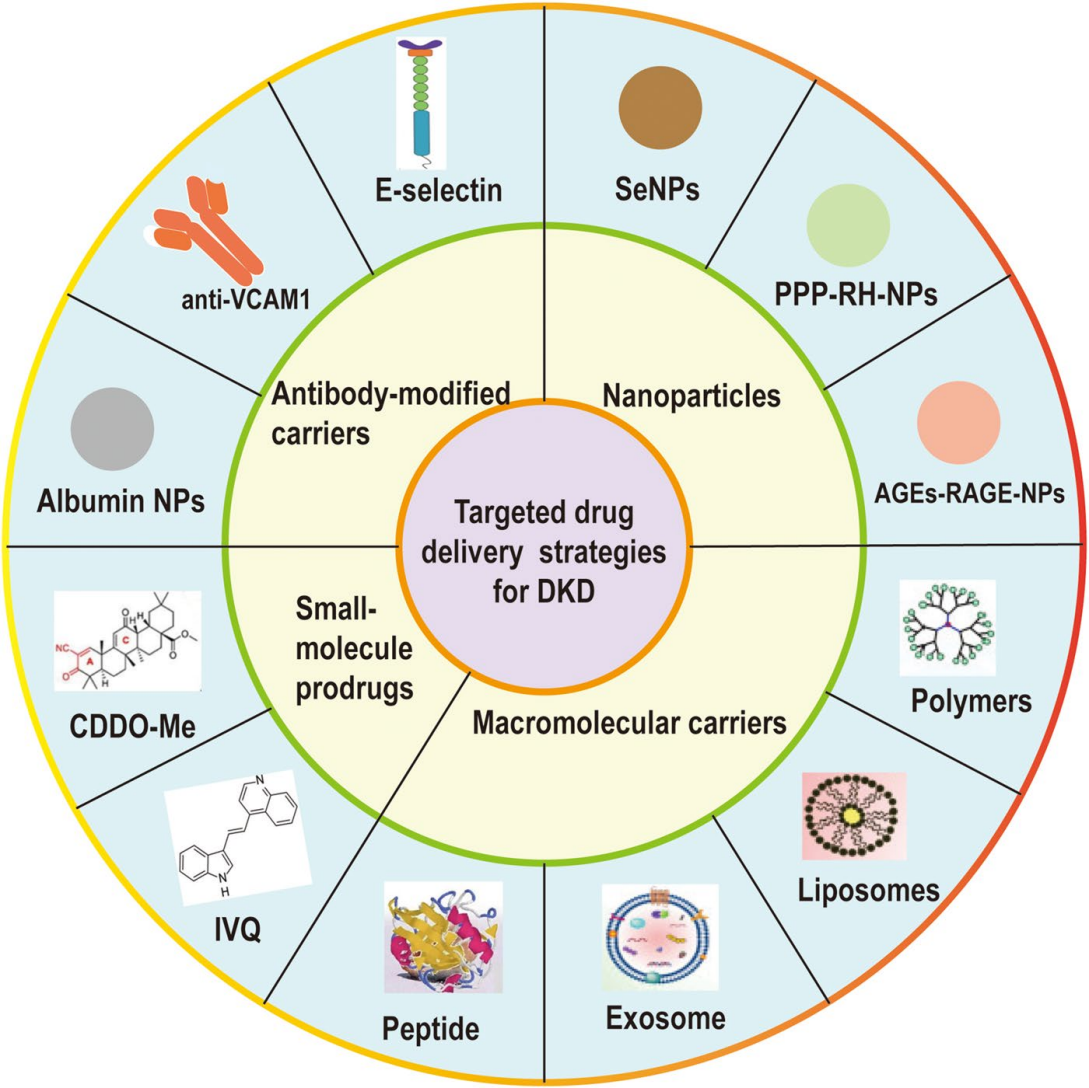
Targeted drug delivery strategies for diabetic kidney disease (DKD).

The targeted treatments for DKD are the research hotspot at present. We mainly review the delivery system about the carriers which can transfer the effective drugs in the therapy of DKD (Figure 2).

### Nanoparticles

**Figure 3.**
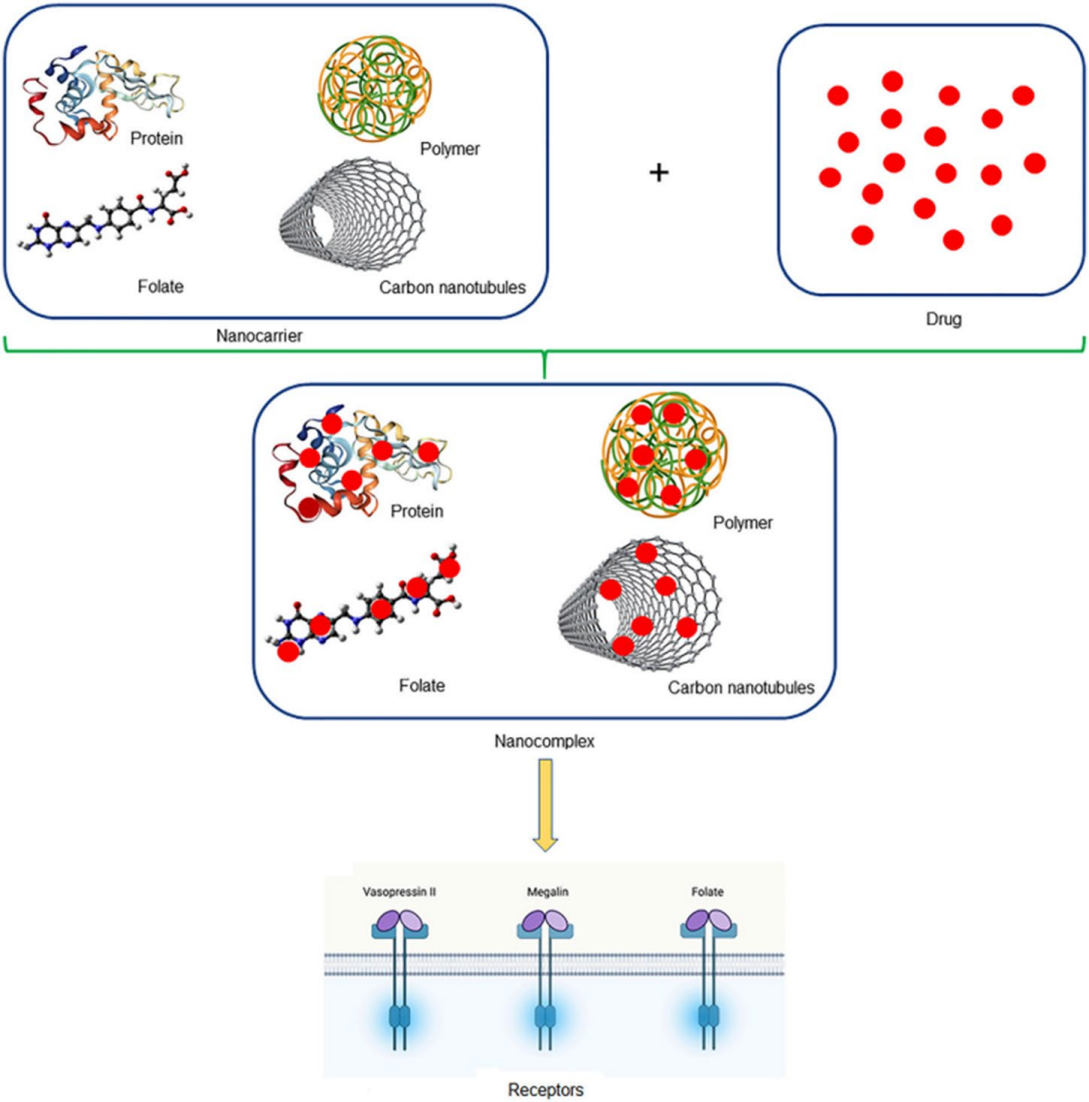
Schematic illustration of nanoparticles (Merlin and Li, 2021).

Nanoparticle is a kind of structure with the size vary from 10–1000 nm, with a solid core surrounded by suitable chemicals which may affect the size and polarity (Xu et al., 2018). The size of the nanoparticles has important effects on circulation half-life, cellular absorption and target cells, and the ones with the size of 100 nm have the longest half-life (Sharma et al., 2011). Many shapes have been constructed such as rod, spherical, cubic, helical and hexagonal, among them the rod one has shown to be more effective and the spherical one clears more slowly (Chauhan et al., 2011; Blanco et al., 2015). The drugs can be encapsulated, dissolved in the nanoparticle or attached to its matrix, which can increase the bioavailability, solubility, drug penetration and protect it from degradation (Qamar et al., 2019). It always combined with chitosan, poly(d,l-lactide-co-glycolide) (PLGA), poly(ɛ-caprolactone) (PCL) polymer in drug delivery area. Figure 3 (Merlin and Li, 2021) briefly summarized the nanocarriers which were used to carry drugs in the form of nanocomplex. Selenium nanoparticles (SeNPs), with the diameter ranging from 40–90 nm, can downregulate the levels of creatine, blood urea nitrogen, collagen and fibronectin and upregulate the albumin level in DKD rats, meanwhile SeNPs regulate the level of Bax and Bcl-2, and elevate the expression of SIRT1 and heat shock protein-70 (Kumar et al., 2014). Rhein (RH) is a kind of Chinese traditional herbal medicine with multi-therapy effects on DKD, but its application is limited by the low bioavailability, poor solubility, decreased renal distribution. RH-loaded polyethyleneglycol-co-polycaprolactoneco-polyethylenimine nanoparticles (PPP-RH-NPs) with the size of 75 ± 25 nm are designed for kidney-targeted drug delivery, which can suppress the expression of TGF-β1 and Smad2/3 phosphorylation and reduce the level of proteinuria, blood creatine, blood urea nitrogen in DKD rats (Chen et al., 2018). Advanced glycation end products (AGEs) and its receptor (RAGE) are potential key therapy targets in DKD. Xuemei Zhou et al have proposed a dual-target nanoparticle (ACEs inhibitors and RAGE inhibitors) with high efficiency and synergistic effect, which may have potential application in the therapy of DKD (Zhou et al., 2012). MET-HMSNs-CeO2, a multifunctional nanoparticle, was doped with ceria in the surface and loaded metformin (MET) in the pore of hollow mesoporous silica nanocomposite (HMSN), which could exhibit antioxidative and antiapoptotic function in DKD (Tong et al., 2020). We summarize the other kinds of nanoparticles in the treatment of DKD (Table 1).

**Table 1.** Nanoparticles in the treatment of DKD.

Nanoparticles	Drug	Size	Experimental model	Function	Reference
HMSN-CeO2	metformin	117 nm	STZ rats NRK-52E cell	antioxidative, antiapoptotic	Y Tong et al. 2020
PEG-b-(PELG-g-PZLL)	quercetin	64 nm	Male SD rats	ameliorate the kidney pathological damage, improve renal function, antioxidative, antiinflammation, downregulated the ICAM-1 expression	Tong et al., 2017
AuNP	pomegranate peel extract (PPE)	20.4 ± 0.347 nm	STZ rats	reduce the fasting blood-glucose, serum urea, creatinine, TC and TG; reduce glomerular sclerosis and renal fibrosis; reduce proinflammatory burden through the axis of MAPK/NF-κB/STAT3/cytokine	Manna et al., 2019
SLN	myricitrin	76.1 nm	STZ-NA rats	reduce oxidative stress, increase antioxidant enzymes level	Ahangarpour et al., 2019
Melanin@Glc-NCM	Glucose ligand	107.5 ± 5.8 nm	SD rats	promote the apoptosis of glomerular MCs and restrain the hyperproliferation of MCs	J Li et al., 2021
KLPPR	rhein	59.5 ± 6.2 nm	STZ rats HK-2 cells	lower serum creatinine and kidney index, improve the creatinine clearance rate by suppressing secretion and accumulation of fibronectin and TGF-beta 1	Wang et al., 2019
Virus-Mimetic NPs	cinaciguat	80 nm	rat-derived MCs	promote anti-Fibrotic effects by suppressing of the TGF-β pathway	Fleischmann et al., 2021
Fucoidan NPs	fucoidan	330.6 ± 58.8 nm	STZ-Wistar rats	antioxidative stress by decreasing MDA and increasing SOD and GPx, anti-inflammatory by decreasing levels of IL-6 and TNF-α.	Wardani et al., 2022

Total cholesterol (TC), triglycerides (TG), solid lipid nanoparticle (SLN), kidney-targeted rhein (RH)-loaded liponanoparticles (KLPPR).

### Macromolecular carriers

#### Polymers

The application of polymers has played a significant role in the targeted drug delivery system. Polymeric micelles, polymersomes and nanohydrogels are polymer nanosystems, assembled by polymers with different compositions, structures and molecular weights, and each type of them has its particular identity to attach or encapsulate therapeutic agents (Joglekar and Trewyn, 2013). They can minimize the adverse effects of drugs by the site-specific therapy. Polymeric micelles, with the dimension of 5–100 nm, can prolong the blood circulation time by reducing the clearance of the reticuloendothelial system and decreasing the drug side effects by lower the opsonization (Joglekar and Trewyn, 2013). Polymersome are polymer-based vesicles with amphiphilic copolymers and it can be stimulated by a certain wide wavelength of light in order to increase the drug efficacy and decrease adverse effects (Hernandez Becerra et al., 2022). Nanohydrogel, possessed the characteristic of hydrogel and nanoparticle, is a three-dimensional and cross-linked hydrophilic polymer network, with the diameter of 1–100 nm (Gonçalves et al., 2010). The nanohydrogel can encapsulate the drug through physical attachment, self-assembly and covalent conjugation (Dalwadi and Patel, 2015). Chen L et al. reported a new one-dimensional Cu (II) coordination polymer by using a NNO tridentate (NNO) Schiff base ligand4-fluoro-2-(((2-(methylamino)ethyl)amino)methyl)phenol, which can downregulate the serum amyloid A and TNF-α in STZ induced DKD rats (Pan-Pan Lin and Chen, 2021).

#### Chitosan

Chitosan is a kind of polysaccharide derived from natural chitin which exists in the crustacean shells, fungal cell walls, arthropods and insects, with the properties of low toxicity, bioactivity, biocompatibility, biodegradability and structural variability (Kean and Thanou, 2010; Motiei et al., 2017).Chitosan can be degraded by chitosanase and lysozyme to form oligosaccharides and monosaccharides, and then absorbed by the body (Woraphatphadung et al., 2018). Chitosan stabilized nanoparticles (Ch-SeNPs) can downregulate the expression of TNF-α, IL-6, and IL-1β in type 2 DM rats, and Ch-SeNPs combine with metformin can reduce the creatine, proteinuria and urea (Khater et al., 2021). Researchers reported that chitosan oligosaccharides can ameliorate the proteinuria, reverse the kidney pathological changes, reduce the expression of TGF-β1 and fibronectin and the activity of urine N-acetyl-β-D-glucosaminidase (NAG) in STZ-T2DM rat model (Zhang et al., 2018).

#### Liposomes

Liposomes are spherical vesicles with an aqueous nucleus surrounded by concentric phospholipid bilayers (Guimaraes et al., 2021). Liposomes are widely used in targeted drug delivery system on account of their nontoxic and biodegradable. The targeting strategies of liposome can be divided into passive and active targeting, while the passive one always relies on the vasculature and the active targeting depends on the binding of the receptor-specific ligands on the liposome surface (Lehner et al., 2013). Quercetin is a sort of bioflavonoid with the features of anti-inflammatory, anti-diabetes, anti-allergic, anti-cancer and reduce aldose reductase (Shi et al., 2019). The poor water solubility of quercetin restrains its application; hence, quercetin liposomes are designed to improve its solubility. Quercetin liposomes significantly downregulate the expression of creatine, urea, TNF-α, IL-1β, AGEs and alleviate the renal histopathological changes (Tang et al., 2020). Oxidative stress plays an important role in the pathogenesis of DKD (Vodosek Hojs et al., 2020). Glutathione is a kind of antioxidant substance, but it is unstable and cannot easily penetrate the cell membrane. Huajuan Shen et al reported glutathione liposomes can improve the stability, bioavailability and antioxidant ability of glutathione, which can target kidney and reduce the proteinuria, creatine, urea, alleviate the kidney fibrosis in DKD rats (Shen and Wang, 2021).

#### Exosomes

Exosomes, with a diameters of 30–150 nm, are extracellular vesicles composed of bilayer membranes, and take part in the process of immune response, inflammation, coagulation, cell proliferation and cell migration (Simons and Raposo, 2009). Exosomes can reduce immunogenicity, enhanced tissue penetration and cross biological barriers, such as the blood–brain barrier and the gastrointestinal tract (Shao et al., 2020). A wide range of molecules, such as drugs, nucleic acid and protein, can be delivered by exosomes in terms of incubation, electroporation or sonication (Gutierrez-Millan et al., 2021). The exosomes can fuze with the targeted cells and release the contents into them. Mesenchymal stem cell (MSC)-derived exosomes downregulate serum creatinine, blood urea nitrogen and proteinuria, decrease mesangial expansion and collagen fibers around glomerular capillaries and the tubules, with obvious upregulating of LC3 and Beclin-1 and downregulating of mTOR, indicating that autophagy induced by exosomes may attenuate the clinical and pathological manifestation of DKD in the STZ-DM rats (Ebrahim et al., 2018). Exosomes, derived from MSC-conditioned medium, are administrated into unilateral kidney subcapsular space of STZ-induced DM rats, which can reduce renal tubules dilatation, tubular epithelial atrophy and inflammatory cells infiltration (Nagaishi et al., 2016). Juan Jin et al reported that adipose-derived stem cells-exosome (ADSCs-Exo) can alleviate the levels of proteinuria, serum creatinine, blood urea nitrogen and podocyte apoptosis in DKD rats, and the underlying mechanism could be the upregulated expression of miR-486 induce the inhibition of the Smad1/mTOR signaling pathway (Jin et al., 2019).

#### Peptide-based carriers

Therapeutic peptides are sometimes limited by their poor stability and poor cellular uptake, in this case, the development of cell-penetrating peptide-based drug delivery system can overcome the limitation to some extent. The cell penetrating peptide (CPP), are short peptides with positive charge, contains synthetic and natural CPP, protein transduction domains (PTDs) and membrane-translocating sequences (Wadia and Dowdy, 2002). These CPPs and the drugs they transport are internalized by cells through the endocytic pathway (Deshayes et al., 2005). Therapeutic peptides in DKD mainly include insulin, GLP-1 and analogs, which are administrated parenterally. Delivery of these peptides by oral route has been a research hotspot. The binding of peptides to CPPs is a potential route for the oral administration of these macromolecular drugs (Kristensen et al., 2016). PLGA-GLP-1 nanoparticles conjugated to CPP, then encapsulated with HPMC-polymer and loaded with iDPP4, which formed GLP-1/iDPP4 delivery multifunctional composite system, and the system was reported to have prolonged hypoglycemic effects in DM rats (Araujo et al., 2016). Thiolated polymer is a kind of delivery system of noninvasive peptides and proteins. Thiolated-chitosan-6-mercaptonicotinic acid for oral insulin delivery system can downregulate the serum glucose in rats (Millotti et al., 2014). Peptide can be delivered by microencapsulation which can protect them from being digested by the gastric acid. Oral β-cyclodextrin insulin microparticles had a promising effect in decreasing the blood glucose in DM rats and the hypoglycemic effect was similar to that of subcutaneous injection of insulin (D’Souza et al., 2015). Nanoparticles, liposome and micelles were also reported to be the promising oral peptides delivery system (Sharma et al., 2015; Ismail and Csoka, 2017).

#### Small-molecule prodrugs

The definition of prodrug raised by Albert can best summarize its features, that is ‘Prodrugs are chemicals with little or no pharmacological activity, undergoing biotransformation to a therapeutically active metabolite (Albert, 1958)’. There are two kinds of prodrugs, the bioprecursors and the carrier-linked prodrugs. Bioprecursors, including quinones, nitroarenes, N-oxides and amidoximes, do not have carriers and they can be activated by hydration, while the carrier-linked prodrugs are always esters activated by enzymatic hydrolysis (Testa, 2009). Gluconeogenesis is an important cause of hyperglycemia in type 2 DM. Researchers synthesized a small molecular 4-[2-(1H-indol-3-yl) vinyl] quinoline (IVQ) as a prodrug, which can suppress hepatic gluconeogenesis and ameliorate hyperglycemia without the cardiovascular side effects and genotoxicity in type 2 DM rats (Zhou et al., 2019). Bardoxolone methyl (CDDO-Me) exhibited a promising therapy for diabetic nephropathy in clinical trials, but the cardiac toxicity restrained its application. Therefore, a series of prodrugs, just as Cathepsin B (CTSB)-α-cyano-α, β-unsaturated ketone (CUK)-polyethylene glycol (PEG), were formed, making CDDO-Me without the active CUK part exposure in order to reduce the side effects of the treatment (Liu et al., 2022).

#### Antibody-modified carriers

Antibody, a complex protein-based molecule, is a crucial component in the indirect ways of active targeting. Antibodies vary in molecular weight, and those with molecular weight greater than 150 kDa cannot pass through the glomerular filtration barrier. Antibodies with the molecular weight lower than 50 kDa may be promising moieties in the kidney-targeted delivery system as they pass through the glomerular filtration barrier and can be reabsorbed by the proximal tubular cells (Chen et al., 2020a). Bovine albumin-based NPs with the diameters of 10 nm can selectively target the Fc receptor (FcRn) on the surface of podocytes (Wu et al., 2017). Anti-vascular cell adhesion protein 1 (VCAM-1)-rapamycin-SAINT-O-Somes can delivery rapamycin to the kidney and have little effect on cellular viability in order to reduce the side effect (Visweswaran et al., 2015). E-selectin is overexpressed in highly inflammatory renal environments and is also used as an antibody target for NP kidney targeting (Asgeirsdottir et al., 2008).

### Targeted drug delivery strategy for MCs

#### Nanoparticles

Glomerular MCs is the potential drug delivery target cell in DKD. Nanoparticles pass through the fenestrated endothelium and get close to the MCs, in this case, the MCs-targeted nanoparticle should be designed to be smaller than the diameter of the endothelium pore. The diameter of glomerular endothelium pore is reported to be 80–100 nm (Luft et al., 1982). PEGylated gold nanoparticles with the size of 75 ± 25 nm can specifically target the MCs in mice (Choi et al., 2011).Chitosan improve the protection efficiency for siRNA and Emine Salva et al construct siPDGF-B- and siPDGFR-β-containing chitosan nanoparticles which can target the MCs to knock down PDGF-B and its receptor in order to decrease the MCs’ proliferation (Salva et al., 2017). Glucose transporter 1(GUT1) is a glucose transporter enriched in the MCs of DKD patients and the expression of GUT1 in MCs is positively correlated with oxidative stress to promote the progression of DKD (Zhang et al., 2010). Astaxanthin (AST) is a natural nontoxic lutein carotenoid with powerful antioxidant capacity (Sila et al., 2015). As the low stability and solubility of AST restrict the antioxidant capacity, AST-GLU-LIP is constructed to overcome the shortcomings and the results demonstrate that AST-GLU-LIP can improve the bioavailability and antioxidant capacity of AST and ameliorate the kidney pathological lesion in DKD rats (Chen et al., 2020b). A kind of glucose ligand modified neutrophil-like cell membrane coated melanin nanoparticle (Melanin @Glc-NCM) was prepared and applied in DKD of gestational rats, which can promote the apoptosis of glomerular MCs and restrain the hyperproliferation of MCs (J Li et al., 2021).

#### Liposome

Thy 1.1 antigen is specifically expressed in MCs. OX7-coupled liposome was equipped by connecting them with the Fab fragments of OX7 monoclonal antibody to thy1.1 antigen, and the OX7-LIP exhibits the specific targeting to MCs in rats (Tuffin et al., 2005). Anti-α8 integrin immunoliposome was a specific delivery to the MCs as the α8 integrin was expressed exclusively in MCs (Scindia et al., 2008). Triptolide (TP) suppresses the proliferation of MCs in DKD via the inhibition of PDK1/Akt/mTOR Pathway (Han et al., 2017). The side effects of TP, such as bone marrow suppression, liver function damage, and genital system toxicity, limit its application. TP was loaded into the TRX-20 modified liposomes and modified with PEG5000, forming PEG-TRX-TP-LP and specific binding to MCs, which displayed anti-inflammatory actions (Yuan et al., 2017). Puerarin is an anti-hyperglycemic and anti-oxidative active ingredient, but the aqueous solubility and poor oral availability restrain its clinical use. Puerarin liposome with the concentration of 50–100 µM was the most effective one to lower the MCs proliferation in rat MCs line under high glucose environment (Barro et al., 2021). Wei B et al reported that quercetin liposome (Q-PEGL) could improve the renal related biochemistry and pathological changes in the STZ induced DKD rats (Tang et al., 2020).

### Targeted drug delivery strategy for podocytes

#### Nanoparticles

Podocytes locate on the outer side of the glomerular capillaries and they are reachable for the drug delivery carriers. Celastrol (CLT) is one of the active ingredients in tripterygium wilfordii, which have therapeutic effect in kidney disease but with organ toxicity. Wu et al designed a kind of peptides coupled CLT-phospholipid lipid nanoparticles (PC-PLNs) to deliver CLT to the podocytes and they can alleviate inflammation and reduce the CLT toxicity in chronic kidney disease (Wu et al., 2022). HDAC4 involves in the process of podocytes injury in DKD and HDAC4 siRNA has revealed a good prospect. DTsiANp/HDAC4 is designed to delivery HDAC4 siRNA to the podocytes in DKD rat model, after four weeks treatment, the glucose level, urinary albumin excretion ratio (UAER), MCs proliferation and glomerular sclerosis are all reduced significantly (Raval et al., 2019). Gold nanoparticles with the diameter of 50 nm can reduce the 24 h urinary albumin excretion rate, blood glucose, GBM thickness and foot process width in DKD rat models (Alomari et al., 2020). CoenzymeQ10 (CoQ10) is an antioxidant and may be a promising treatment for early-stage DKD. However, the low water solubility and nonspecific distribution of coenzyme Q10 limit its clinical application. In this condition, liposomes containing CoQ10 (CoQ10-lip) are equipped and combined with ultrasound microbubbles in DKD rat models, with a result of improved proteinuria and oxidative stress indexes (Yue et al., 2017).

#### Peptide

Inflammation involves in the pathogenesis of DKD. NF-κB is a bridge linking inflammation and hyperglycemia. A Cell-permeable peptide, designing with the inhibitor of kappa B kinase γ (IKKγ)/NF-κB essential modulator (NEMO)-binding domain (NBD), alleviates the albuminuria, kidney damage, podocyte loss and GBM thickness in DKD rat models (Opazo-Rios et al., 2020).

#### Polymers

miRNA30a, which is primarily responsible for podocyte homeostasis, is suppressed by hyperglycemic in DKD, leading the apoptosis of podocyte. Therefore, delivery exogenous miRNA30a to podocyte may ameliorate the podocyte injury. Cyclo (RGDfC)-gated polymeric-nanoplexes with dendrimer templates are can transfer the miRNA30a to the podocytes and alleviate the injury of podocytes in DKD (Raval et al., 2021).

### Targeted drug delivery strategy for renal tubular epithelial cells (RTECs)

#### Nanoparticles

RETCs are vital target cells in the kidney targeted drug delivery systems for preventing renal fibrosis of DKD. PLGA-Gypenoside (Gyp) XLIX nanoparticles can effectively reduce collagen deposition and inhibit renal fibrosis in RETCs (HK2) cells stimulated by TGF-β (Q Liu et al., 2021). FITC labeled renal tubular-targeting peptide modified PLGA-PEG nanoparticles are used to transfer asiatic acid, which exhibits renal protective and anti-fibrosis effects (He et al., 2020). Pax2 gene, associating with interstitial fibrosis, is expressed in RTECs, and polyethyleneimine nanoparticles are employed to deliver Pax2-siRNA to RTECs, which can ameliorate the tubular damage and interstitial fibrosis (Li et al., 2012). Autophagy has been reported to be involved in the albuminuria caused renal tubular injury, and Fe_3_O_4_ magnetic nanoparticles are related to the autophagy. Fe_3_O_4_ magnetic albumin nanoparticles can alleviate renal tubular injury and delay the development of tubulointerstitial fibrosis (L Liu et al., 2021).

#### Liposome

Heat shock protein 72 (HSP72) is a stress-inducible protein capable of protecting cells from ischemic injury. Liposomes containing HSP72 are transferred in the renal tubular cells (LLC-PK1), which prevent the activation and translocation of NF-κB, and the apoptosis of renal tubular cells (Meldrum et al., 2003).

## Discussion and conclusion

We conclude the advantages and disadvantages of different kinds of targeted drug delivery systems in Table 2. At present, researches on targeted drug delivery systems for DKD are almost in the early stages, and most of them have significant therapeutic effects and reduced side effects in animal models or cell series. But there are still several limitations. The first is biosecurity, as the toxicity of most non-biodegradable carriers is unclear. The second is the clinical efficacy. Most of the research data is obtained from animals and cells, which is different from the actual patients. The third is the stability of the transporter. Producing large-scale stable high-quality NPs and liposomes remains difficult. In this circumstance, targeted drug delivery systems with nontoxic, biodegradable and non-immunogenic characteristics may be facilitate by the transition from laboratory to clinical application.

**Table 2.** The advantages and disadvantages of different kinds of targeted drug delivery systems.

	Advantages	Disadvantages
Nanoparticles	Increase the bioavailability, solubility, drug penetration and protect it from degradation (Qamar et al., 2019)	burst release during circulation in the blood, low targeting efficacy, and toxicity (Park and Na, 2015)
Polymers	Prolong the blood circulation, decreasing the drug side effects (Joglekar and Trewyn, 2013)	low solubility at physiological pH and rapid drug release in the aqueous medium (Farshbaf et al., 2018)
Liposomes	Nontoxic and biodegradable	Interact with serum proteins lead to destabilization and rapid clearance (Gregoriadis and Perrie, 2010)
Exosomes	Reduce immunogenicity, enhanced tissue penetration, and Cross biological barriers (Shao et al., 2020)	scale-up production yield and control of purity is difficult (Shao et al., 2020)
Peptide-based carriers	Better biocompatibility, biochemical and biophysical properties, lack of toxicity, controlled molecular weight (Berillo et al., 2021)	induce side effects as the artificial nature and lack of ability to penetrate the blood-brain barrier (Berillo et al., 2021)
Small-molecular prodrugs	Low cost, easy preparation, and stable storage (G Li et al., 2021)	narrow therapeutic window and unfavorable pharmacokinetic properties (G Li et al., 2021)
